# ORAL CARE IS A NECESSITY NOT A LUXURY: POLICIES THAT HELP OR HINDER ORAL CARE FOR OLDER ADULTS

**DOI:** 10.1093/geroni/igae098.2489

**Published:** 2024-12-31

**Authors:** Kayla Gallant, Katrina Reid, Joan Ilardo, Angela Zell

**Affiliations:** Michigan State University College of Human Medicine - East Lansing, East Lansing, Michigan, United States; Michigan State University East Lansing, East Lansing, Michigan, United States; Michigan State University East Lansing, East Lansing, Michigan, United States; Michigan State University East Lansing, East Lansing, Michigan, United States

## Abstract

We interviewed fifteen stakeholders including state-level policy officials, medical practitioners, dental advocacy organizations, and educators to identify factors influencing oral care provision for older adults in residential settings. The interviews were conducted by two second-year medical students under the supervision of a Ph.D. social worker and a Doctor of Public Health. Analysis of interview transcripts followed grounded theory methodology. Our findings revealed several key themes limiting the provision of dental care to older adults in residential settings. These themes included financial constraints (e.g., Medicare and Medicaid coverage and reimbursement could be unpredictable state by state and year by year), inadequate training (e.g., few specialized dental training programs addressing geriatric oral care needs), limited resources for care provision within nursing facilities, and a systemic insufficient awareness of the dental needs of older adults in residential settings. This study’s results illustrate the means of overcoming the limitations plaguing oral care for older adults in residential settings and ultimately advancing geriatric oral health outcomes in skilled nursing facility (SNF) settings. Next steps are to introduce a “train-the-trainer” model at a SNF. Registered and student dental hygienists will train certified nursing assistants to provide basic oral care for older residents in the SNF to improve geriatric oral health, ensure access to basic oral care, and strengthen the SNF’s ability to provide dental services.

